# Adolescents and female patients are at increased risk for contralateral anterior cruciate ligament reconstruction: a cohort study from the Swedish National Knee Ligament Register based on 17,682 patients

**DOI:** 10.1007/s00167-017-4517-7

**Published:** 2017-03-15

**Authors:** Thorkell Snaebjörnsson, Eric Hamrin Senorski, David Sundemo, Eleonor Svantesson, Olof Westin, Volker Musahl, Eduard Alentorn-Geli, Kristian Samuelsson

**Affiliations:** 1 Department of Orthopaedics, Institute of Clinical Sciences, The Sahlgrenska Academy, University of Gothenburg, Göteborg, Sweden; 2 Department of Orthopaedics, Sahlgrenska University Hospital, Mölndal, Sweden; 3 Department of Health and Rehabilitation, Institute of Neuroscience and Physiology, The Sahlgrenska Academy, University of Gothenburg, Göteborg, Sweden; 4 Department of Orthopaedic Surgery, University of Pittsburgh School of Medicine, Pittsburgh, PA USA; 5 Fundación García-Cugat, Barcelona, Spain; 6 Artroscopia GC, SL, Barcelona, Spain; 7 Mutualidad Catalana de Futbolistas-Delegación Cataluña, Federación Española de Fútbol, Barcelona, Spain; 8 Department of Orthopaedic Surgery, Mayo Clinic, Rochester, MN USA

**Keywords:** Knee, Contralateral, Anterior cruciate ligament, ACL, Surgical predictors, Autograft, Registry

## Abstract

**Purpose:**

The impact of different surgical techniques in index ACL reconstruction for patients undergoing contralateral ACL reconstruction was investigated.

**Methods:**

The study was based on data from the Swedish National Knee Ligament Register. Patients undergoing index ACL reconstruction and subsequent contralateral ACL reconstruction using hamstring graft under the study period were included. The following variables were evaluated: age at index surgery, gender, concomitant meniscal or cartilage injury registered at index injury, transportal femoral bone tunnel drilling and transtibial femoral bone tunnel drilling. The end-point of primary contralateral ACL surgery was analysed as well as the time-to-event outcomes using survivorship methods including Kaplan–Meier estimation and Cox proportional hazards regression models.

**Results:**

A total of 17,682 patients [*n* = 10,013 males (56.6%) and 7669 females (43.4%)] undergoing primary ACL reconstruction from 1 January 2005 through 31 December 2014 were included in the study. A total of 526 (3.0%) patients [*n* = 260 males (49.4%) and 266 females (50.6%)] underwent primary contralateral ACL reconstruction after index ACL reconstruction during the study period. Females had a 33.7% greater risk of contralateral ACL surgery [HR 1.337 (95% CI 1.127–1.586); (*P* = 0 0.001)]. The youngest age group (13–15 years) showed an increased risk of contralateral ACL surgery compared with the reference (36–49) age group [HR 2.771 (95% CI 1.456–5.272); (*P* = 0.002)]. Decreased risk of contralateral ACL surgery was seen amongst patients with concomitant cartilage injury at index surgery [HR 0.765 (95% CI 0.623–0.939); (*P* = 0.010)]. No differences in terms of the risk of contralateral ACL surgery were found between anatomic and non-anatomic techniques of primary single-bundle ACL reconstruction, comparing transportal anatomic technique to transtibial non-anatomic, anatomic and partial-anatomic.

**Conclusion:**

Age and gender were identified as risk factors for contralateral ACL reconstruction; hence young individuals and females were more prone to undergo contralateral ACL reconstruction. Patients with concomitant cartilage injury at index ACL reconstruction had lower risk for contralateral ACL reconstruction. No significant differences between various ACL reconstruction techniques could be related to increased risk of contralateral ACL reconstruction.

**Level of evidence:**

Retrospective Cohort Study, Level III.

## Introduction

Recent research has focused on anterior cruciate ligament (ACL) reconstruction and performed with various surgical techniques [16]. Patients with a previous ACL reconstruction and later suffering from a contralateral ACL injury have an uphill battle to recapture their pre-injury level of activity. Reports have described the incidence of contralateral ACL injury from 2 to 15% [1, 3, 8, 21] and typically occurs 1–4 years after the index surgery [18, 21]. These numbers can vary depending on the length of follow-up. To be able to maximise the probability of successful ACL reconstruction, it is very important to acknowledge possible risk factors. Previous studies have identified young age [2] and return to high activity level sports [7, 12] as risk factors for contralateral ACL reconstruction.

In recent years, different surgical techniques for ACL reconstruction have been used. However, there is insufficient knowledge in regard to the association of surgical techniques and contralateral ACL reconstruction. With increased data registration, more information about risk factors for contralateral ACL reconstruction has become available, creating better opportunities for such analyses and may have implications for treatment of these patients.

The purpose of this population-based cohort study was to investigate risk factors for contralateral ACL reconstruction, based on analysis of data from the Swedish National Knee Ligament Register (SNKLR).

## Materials and methods

Patient data were extracted from the SNKLR. Patients registered for index (primary unilateral) ACL reconstruction from 1 January 2005 to 31 December 2014 and registered to undergo contralateral ACL surgery were included, excluding patients with a follow-up shorter than the earliest documented event in the specific cohort. Patients aged 13–49 at the time of the index reconstruction, who underwent reconstruction with hamstring tendon (HT), were assessed for eligibility. All data on surgical technique were acquired with online questionnaire, launched in January 2015 and sent to all surgeons in Sweden performing ACL reconstruction during the study period. This questionnaire was designed specifically to collect detailed information about surgical technique among orthopaedic surgeons in Sweden performing ACL reconstructions. This information was collected retrospectively and has been reported in a previous study [5]. Patients were excluded from the study if they were not of age between 13 and 49 years of age, had concomitant fracture, nerve injury, vascular injury, ligament injury requiring repair/reconstruction, not operated with hamstrings graft or their data had incomplete information.

### The Swedish National Knee Ligament Register

The SNKLR is a nationwide population-based database, initiated in 1 January 2005. The database is one of three Scandinavian knee ligament registries. Data are collected prospectively on ACL injuries and associated knee surgery, and it is estimated that the SNKLR covers more than 90% of all ACL operations in Sweden [14]. The patient-unique Swedish social security number is used to identify patients. The internet-based protocol consists of patient-reported section and surgeon-reported section. It is the responsibility of the surgeon to register information about activity level at the time of the injury, time from injury to reconstruction, graft selection and fixation techniques. Moreover, data are registered including information about other injuries to the knee, prior surgeries to the index knee or the contralateral knee along with information about treatments such as meniscal or chondral injuries.

When the patient undergoes revision surgery or reoperation by any means, the surgeon registers this as separate entries and the event is correlated to the index ACL reconstruction.

Extracted data from the database are anonymous and investigators only have access to unidentifiable patient data.

### Investigated variables

The following variables were evaluated: age at index surgery, gender, concomitant meniscal or cartilage injury registered at index injury, visualisation of known surgical landmarks at index reconstruction, transportal femoral bone tunnel drilling and transtibial femoral bone tunnel drilling (including anatomic, non-anatomic and partial-anatomic drilling).

### Outcome measures

The study end-point was a primary ACL reconstruction of the contralateral knee during the 10-year follow-up.

### Ethics

This cohort study was performed with data from SNKLR. Participation in the SNKLR is voluntary for both patients and surgeons. Consent is not mandatory for research work in national databases in Sweden. During the research work, access was only given to unidentifiable patient data. The Regional Ethical Review Board in Gothenburg, Sweden approved the research (ID 760-14).

### Statistical analysis

Tables and diagrams were generated using Microsoft Excel for Windows (Version 14.0.7, Microsoft Corp, Redmond, WA, USA). A statistician assigned to the SNKLR performed all statistical analyses. Statistical analysis was performed in IBM SPSS Statistics (Version 23.0, IBM Corp, Armonk, NY, USA). The data were summarised using counts and percentages for descriptive data and means ± SDs and median and range for patient-reported outcome data. The end-point of contralateral ACL surgery was analysed as time-to-event outcomes using survivorship methods including Kaplan–Meier estimation and Cox proportional hazards regression. All survival estimates and hazard ratios (HRs) were reported with 95% CIs. Statistical significance was defined as a 95% CI for hazard ratios not including 1.0 and a *P* value <0.05. Additionally, a multivariate analysis adjusted for possible confounding factors (patient sex, age at index ACL reconstruction and concomitant injury to meniscus or cartilage) was performed using a Cox proportional hazards regression expressed as HR and 95% confidence intervals (CI). No minimum follow-up time was pre-specified; instead patients with a shorter follow-up than the earliest documented event (contralateral ACL surgery) in the specific cohort were censored from analysis.

## Results

**Fig. 1:**
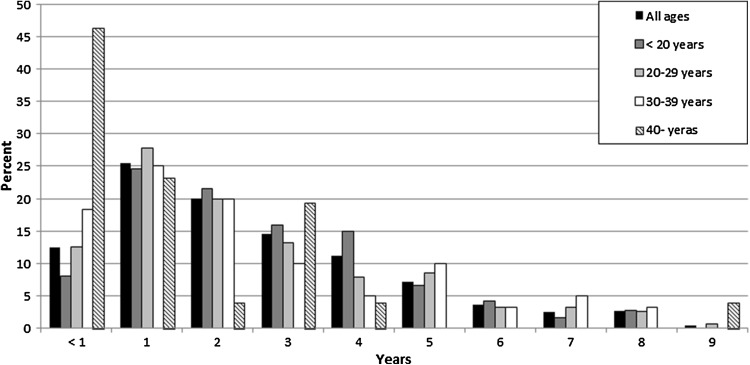
Yearly proportion of contralateral anterior cruciate ligament reconstruction after index surgery

A total of 17,682 patients were included in the study [*n* = 10,013 males (56.6%) and 7669 females (43.4%)] (Table 1). Response rate of questionnaire from a total of 108 surgeons was 61.7%. The median age at index surgery was 24 years (range 13–49 years). From the 1st of January 2005 to the 31st of December 2014, a total of 526 (3.0%) patients were reported to undergo contralateral ACL surgery [*n* = 260 males (49.4%) and 266 females (50.6%)] (Table 2). The proportion of contralateral ACL reconstructions, unadjusted for the length of follow-up, is presented in Fig. 1.

**Table 1:** Characteristics of baseline cohort

Cohort (*n* = 17,682)		
	%	Number
Gender		
Male	56.6	10,013
Female	43.4	7669
Age at index ACL reconstruction (years)		
13–15	7.4	1300
16–20	28.7	5057
21–25	20.7	3667
26–30	14.2	2513
31–35	10.0	1777
36–49	18.9	3350
Meniscus injury present (medial and/or lateral) at index surgery		
Yes	43.8	7743
No	56.2	9939
Cartilage injury present at index surgery		
Yes	26.0	4598
No	74.0	13,084
Meniscus and/or cartilage injury at index surgery		
Yes	54.8	9685
No	45.2	7997

**Table 2:** Gender, age and concomitant injury and risk of contralateral ACL reconstruction

	Contralateral ACL reconstruction cohort (*n* = 526)				
	%	*N*	Hazard rate	95% CI	*P* value
Gender					
Male^a^	49.4	260	1.337	1.127–1.586	0.001
Female	50.6	266			
Age at index ACL reconstruction (years)					
13–15	16.7	88	2.771	1.456–5.272	0.002
16–20	43.7	230	1.230	0.689–2.197	n.s.
21–25	16.7	88	0.752	0.394–1.441	n.s.
26–30	8.2	43	0.491	0.229–1.054	n.s.
31–35	6.1	32	0.532	0.232–1.220	n.s.
36–49^a^	8.6	45			
Meniscus injury present (medial and/or lateral) at index surgery					
Yes	45.1	237	1.086	0.914–1.289	n.s.
No^a^	54.9	289			
Cartilage injury present at index surgery					
Yes	22.4	118	0.765	0.623–0.939	0.010
No^a^	77.6	408			

*ACL* anterior cruciate ligament, *CI* confidence interval

^a^Reference group

### Gender

Females had a greater risk of contralateral ACL surgery ([HR] 1.337 [95% CI, 1.127–1.586]; *P* = 0 0.001) (Table 2).

### Age at index ACL reconstruction

The patients were stratified in six distinctive age groups (Tables 1, 2). The oldest age group (36–49 years) was set as the reference group and hence all subsequent comparisons were made to that group. Only the youngest age group (13–15 years) showed an increased risk of contralateral ACL surgery compared with the reference age group [HR 2.771 (95% CI 1.456–5.272); *P* = 0.002]. The other stratified age groups were not associated with the risk of contralateral ACL surgery when compared with the reference group (36–49 years) (Table 2).

### Meniscus injury and cartilage injury

A concomitant meniscus injury at index ACL surgery was not associated with the risk of contralateral ACL surgery [HR 1.086 (95% CI 0.914–1.289); *P* = n.s.] (Table 2). However, a decreased risk of contralateral ACL surgery was seen amongst patients with concomitant cartilage injury at index surgery [HR 0.765 (95% CI 0.623–0.939); *P* = 0.010] (Table 2).

### Surgical technique

**Fig. 2:**
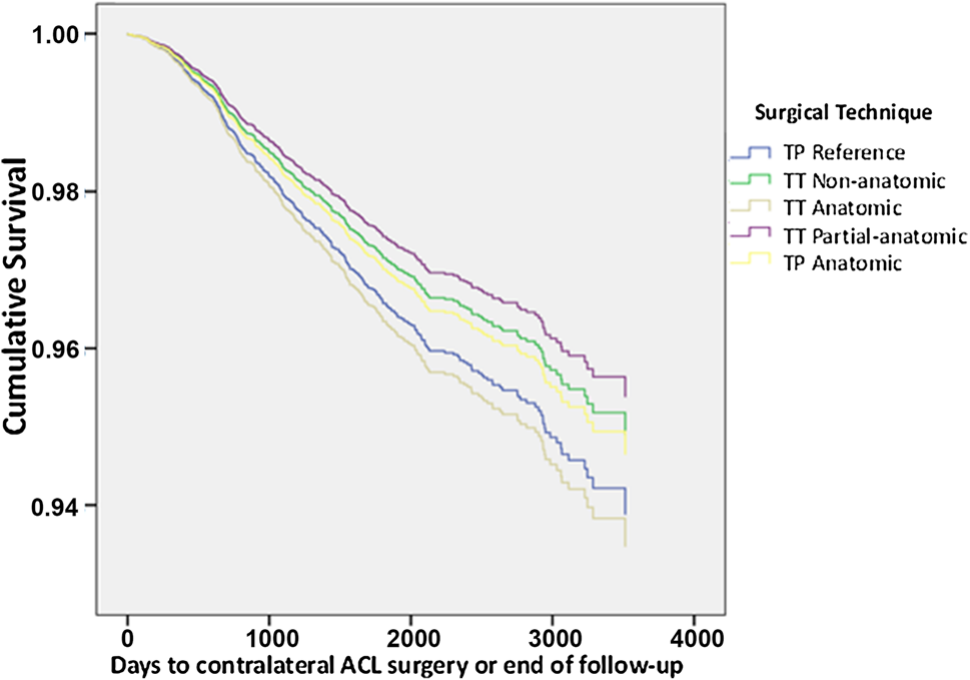
Kaplan–Meier survival function of surgical technique and contralateral ACL surgery

No differences in the risk of contralateral ACL surgery were found between the surgical techniques of primary single-bundle ACL reconstruction when transportal (TP) anatomic technique was used as reference (Table 3; Fig. 2).

**Table 3:** Surgical technique and risk of contralateral ACL reconstruction

Group				HR			Adjusted HR^a^		
				HR	95% CI	*P* value	HR	95% CI	*P* value
Comparison group	No. of events^b^	Reference group	No. of events^b^						
TP reference (*n* = 6685)	*n* = 153	TP anatomic (*n* = 4036)	*n* = 99	1.159	0.899–1.493	0.254	1.147	0.890–1.478	n.s.
TT non-anatomic (*n* = 1296)	*n* = 54			0.957	0.686–1.337	0.789	0.951	0.681–1.329	n.s.
TT anatomic (*n* = 2158)	*n* = 96			1.241	0.936–1.645	0.134	1.226	0.925–1.626	n.s.
TT partial-anatomic (*n* = 1516)	*n* = 55			0.828	0.594–1.154	0.265	0.859	0.617–1.198	n.s.
All landmarks (*n* = 9397)	*n* = 246	No landmarks (*n* = 831)	*n* = 41	1.144	0.815–1.604	0.437	1.152	0.821–1.617	n.s.
Both footprints (*n* = 14,236)	*n* = 401	No footprints (*n* = 1270)	*n* = 53	1.226	0.918–1.637	0.167	1.250	0.936–1.669	n.s.
Both ridges (*n* = 10,576)	*n* = 276	No ridges (*n* = 3173)	*n* = 111	1.157	0.925–1.446	0.201	1.145	0.915–1.432	n.s.
TP Drilling (*n* = 12,440)	*n* = 314	TT Drilling (*n* = 5109)	*n* = 208	1.034	0.866–1.236	0.709	1.034	0.865–1.236	n.s.

*CI* confidence interval, *HR* hazard ratio, *TP* transportal, *TT* transtibial

^a^Multivariate Cox regression analysis adjusted for patient sex, patient age and meniscal or chondral injury

^b^Event = contralateral ACL surgery

### Surgical factors

The visualisation of all landmarks, visualisation of both footprints, visualisation of both ridges and transportal femoral bone tunnel drilling were not associated with the risk of contralateral ACL surgery (Table 3).

## Discussion

This population-based cohort study covered the time span of 10 years and included a total of 17,682 patients. The most important findings in this study were that a younger age at index ACL reconstruction and female sex were the risk factors for contralateral ACL reconstruction and that the different surgical variables that were investigated could not shed light on any significant difference in outcome. Overall, the contralateral ACL reconstruction rate was 3.0%, similar to prior studies [15]. It is also noteworthy that a decreased risk of contralateral ACL reconstruction was seen amongst patients with concomitant cartilage injury at the time of index ACL reconstruction. The end-point was contralateral ACL reconstruction but no minimum follow-up time was pre-determined. The patients that had a shorter follow-up than the earliest documented event (contralateral ACL surgery) were censored from the analysis.

### Gender

Female patients had 33.7% greater risk of contralateral ACL reconstruction. It is interesting that previous long-term studies [15] have reported no association between patient sex and contralateral ACL injury [7] or a trend of higher risk for females [10]. A recent 15-year follow-up study including patients younger than 18 years of age when undergoing index ACL reconstruction has even recognised higher risk of contralateral ACL injury for males [11]. Previous studies such as Shelbourne et al. [13] have reported a higher rate of contralateral ACL injury for women with 5-year follow-up. Of course, it is important to acknowledge that contralateral ACL injury is not the same as contralateral ACL reconstruction.

### Patient age

In the present study, only the youngest age group (13–15 years of age) showed increased risk of contralateral ACL rupture, using the oldest age group (36–49 years of age) as a reference group. This is in accordance with prior studies [10, 19]. There are several contributing factors to these numbers. This may be the result of higher activity level of younger patients, both pre- and post-operatively. Eagerness to return to competitive sport as soon as possible could also be a contributing factor. Biological and genetic factors [4] could be involved, for example, patients that are genetically prone to ligament injuries might sustain such injuries at a young age [11, 20].

### Meniscal and chondral injuries

Meniscal injury at index ACL reconstruction was not associated with increased risk of contralateral ACL reconstruction. Patients with cartilage injury at index ACL reconstruction had decreased risk of contralateral ACL reconstruction. It is noteworthy to acknowledge that patient’s activity is less likely to return to pre-injury level with more severe injury. These results are in line with prior studies in this field, stating that neither meniscal nor chondral injury at the time of index ACL reconstruction predicted increased risk of contralateral ACL reconstruction [12].

### Surgical technique

In the study, all surgical data were collected from online questionnaire. Patients were then divided into groups depending on surgical approach described by the operating surgeon. Transportal anatomic technique was used as reference method. No statistically significant difference between various surgical approaches and risk of contralateral ACL reconstruction could be seen. Additionally, no difference in risk for contralateral ACL reconstruction could be seen by visualising known anatomical landmarks. Prior studies have indicated that non-anatomic bone tunnel placement via transtibial drilling has the lowest risk of revision surgery after ACL reconstruction [5]. Despite prior studies focusing on various forces in and around the knee [6] with different surgical approaches [9], no significant risk factor for contralateral ACL reconstruction has been identified in this study. This could partly be explained by other contributing factors such as graft size, patient’s activity level, surgeons’ learning curve or evolution of operating methods during the study period.

A limitation of this study is that the primary end-point is contralateral ACL surgery and not contralateral ACL injury. According to the entries in the SNKLR database, it was only possible to include patients that underwent operation for contralateral ACL rupture. Patients that have chosen non-surgical treatment or are not eligible for surgery are therefore not included in our numbers. With older age, considerable number of patients either reduces their activity level or does not proceed to operation because of long and cumbersome rehabilitation bearing in mind their previous experience. Another important limitation is the fact that the surgical data were collected from online questionnaire and retrospective analysis was performed, hence creating risk for recall bias. Only patients operated with hamstrings graft were included in the study, conclusions should be made bearing that in mind. Important additional factors that could significantly influence our results include activity level before and after surgery, the surgeons’ previous experience, postoperative rehabilitation protocol and compliance. The results of the present suggest that younger and female patients may be at increased risk of contralateral ACL reconstruction. This may be view as additional support for targeted neuromuscular training interventions for primary and secondary prevention of ACL injuries, which previously has been shown to be beneficial to reduce the risk of ACL injuries in young athletes [17].

## Conclusion

Increased risk for contralateral ACL rupture was associated with younger age. Females have an increased risk for contralateral ACL surgery compared to males and concomitant cartilage injury at the time of index ACL was associated with the risk for contralateral ACL surgery. Surgical technique does not have decisive influence on the risk of contralateral ACL reconstruction.
